# Inter-Relationship between Platelet-Derived Microparticles and Oxidative Stress in Patients with Venous Thromboembolism

**DOI:** 10.3390/antiox9121217

**Published:** 2020-12-02

**Authors:** Salvatore Santo Signorelli, Gea Oliveri Conti, Maria Fiore, Maria Grazia Elfio, Antonio Cristaldi, Ilenia Nicolosi, Pietro Zuccarello, Luca Zanoli, Agostino Gaudio, Domenico Di Raimondo, Margherita Ferrante

**Affiliations:** 1 Department of Clinical and Experimental Medicine, University of Catania, Via Santa Sofia 87, 95123 Catania, Italy; luca.zanoli@unict.it (L.Z.); agostino.gaudio@unict.it (A.G.); 2 Department of Medical, Surgical and Advanced Technologies “G.F. Ingrassia”, University of Catania, Via Santa Sofia 87, 95123 Catania, Italy; olivericonti@unict.it (G.O.C.); m.fiore@unict.it (M.F.); antonio.cristaldi81@gmail.com (A.C.); ileniatiziananicolosi@yahoo.it (I.N.); pietro.zuccarello@unict.it (P.Z.); marfer@unict.it (M.F.); 3 HCRM Hospital & Clinical Risk Manager Scientific Association, Via Guglielmo Mengarini 88, 00149 Roma, Italy; mariagrazia.elfio@virgilio.it; 4 Division of Internal Medicine and Stroke Care, Department of Promoting Health, Maternal-Infant, Excellence and Internal and Specialized Medicine (Promise) G. D’Alessandro, University of Palermo, 90127 Palermo, Italy; domenico.diraimondo@unipa.it

**Keywords:** oxidative stress, microparticles, biomarkers, platelets, venous thromboembolism, pathophysiology

## Abstract

Background: Hypercoagulative conditions play a key role in venous thromboembolism (VTE). Inflammation is currently linked to VTE, but the potential role of circulating microparticles and oxidative stress (OxS) must be elucidated. The aim of this study was to evaluate platelet-derived microparticles and surrogate OxS biomarkers in patients diagnosed with VTE through a case–control study. Methods: Platelet-derived microparticles (MPs), pro-thrombinase-induced clotting time assay (PiCT), phospholipids (PLPs), malondialdehyde (MDA), 4-hydroxynonenale (4-HNE), thiobarbituric acid reactive substances (TBARs), superoxide dismutase (SOD), and galectin-3 (Gal-3) were measured in VTE patients and in healthy controls. Results: PLPs, 4-HNE, TBARs, and Gal-3 were higher in VTE patients compared to controls; conversely, SOD was lower. A significant non-linear regression between OxS biomarkers and the markers of platelet degranulation was found. Conclusion: Our results suggest that OxS and platelet degranulation are concomitant pathophysiological mechanisms in VTE.

## 1. Introduction

Hypercoagulative conditions play a fundamental role in venous thromboembolism (VTE). Consequently, anticoagulant drugs represent the first-line therapy for treating VTE (class I, level A recommendation) to counteract the abnormal cascade of activated coagulative factors [1]. The role played by platelets (P) in VTE has been less investigated, although their importance in promoting thrombus formation has been elucidated [2,3]. In VTE, there is elevated P aggregability, and many agents and/or conditions (i.e., the environment, inflammatory disease, and malignancy) provoke changes in the reactivity of P. Moreover, activated P characterizes several syndromes (e.g., antiphospholipid syndrome and heparin-induced thrombocytopenia disseminated intravascular coagulation) with a high risk of VTE [4,5,6]. Interestingly, the results from randomized clinical studies have elucidated the role of platelets in VTE, showing a reduced risk with aspirin prophylaxis [7,8,9].

Activated blood cells (i.e., leukocytes, erythrocytes, and platelets) are capable of releasing microparticles (MPs) with strong procoagulative functions. The procoagulant capability of these MPs has been attribuited to tissue factor (TF) and phospholipids in the membrane. In this regard, it is interesting to note that the surface of platelet-derived MPs binds coagulation factors, including coagulative cascade factors [10,11].

Oxidative stress (OxS), defined as the imbalance between the oxidant and antioxidant systems, results from either overproduction of free oxygen radicals or from the insufficiency of antioxidant mechanisms designed to counteract them. The role of OxS in ischemic arterial diseases is well known, but few studies have been performed to elucidate the role of OxS in VTE [12,13] or the validity of biomarkers for the screening of individuals at risk of VTE [14,15]. It has been reported that OxS is higher in patients with VTE [16].

Galectin-3 (Gal-3) contributes to thrombosis through its pro-inflammatory capability, depending on inflammatory mediators such as interleukin-6. Gal-3 is considered a potential marker of endothelial cell dysfunction [17]. Although MPs and OxS have been analyzed separately in previous studies, the present study aimed to assess MPs, surrogate OxS biomarkers, and Gal-3 concentrations in patients with VTE. We planned our study as a case–control study to evaluate the potential correlations. We evaluated MPs, pro-thrombinase-induced clotting time assay (PiCT), phospholipids (PLPs), malondialdehyde (MDA), 4-hydroxinonenale (4-HNE), thiobarbituric acid reactive substances (TBARs) as the sum of MDA and 4-HNE, and the enzyme superoxide dismutase (SOD) as a marker of antioxidant capacity (leukocyte–endothelial cell interactions). Evidence was accumulated regarding the relationship between VTE and inflammation [18,19,20,21].

## 2. Study Population

We carried out a case–control study, enrolling 36 consecutive patients hospitalized in the General Medicine Division (University Hospital G. Rodolico, Catania, Italy) for VTE, paired with a group of control subjects (Table 1). Patients aged over 80 years, with active acute liver disease, chronic renal insufficiency, rheumatic or immunologic disease, or active cancer were excluded from this study. Out of 36 patients, 11 were diagnosed with pulmonary embolism (PE) and 25 with deep vein thrombosis (DVT) of the lower limbs (Table 1). PE was diagnosed by computed tomography (CT) angiography, and DVT was diagnosed by ultrasound examination of the deep veins of the lower limbs; a thrombus visible in the venous lumen with no compression of the vein by the ultrasound probe indicated a positive compression ultrasound (CUS) test. All patients were informed about the research and gave informed consent to participate in the study and for a blood sample to be drawn. The study was conducted according to the Declaration of Helsinki. The study was approved by the Ethics Committee of the Garibaldi Hospital (Catania, Italy; resolution n.23/2016/CECT2).

### 2.1. Laboratory Tests

All patients and controls provided a venous blood sample in the morning, which was fractionated into serum, citrate, and ethylenediamine tetra acetic acid (EDTA) vacuum tubes. We measured MDA, 4-HNE, and TBARs in order to evaluate OxS status and its severity. SOD was measured as a marker of antioxidant capacity (leukocyte–endothelial cell interactions). Gal-3 was also measured as a potential marker of endothelial cell dysfunction. Finally, we also measured platelet-derived MPs and PLPs (µg/mL) and performed the PiCT (s), which are accepted assays to evaluate platelet degranulation.

#### 2.1.1. Platelet-Derived Microparticles (MPs)

Venous blood samples were centrifuged at 1500 rpm for 15 min with an Eppendorf 5417 C/R centrifuge to obtain platelet-poor plasma. A second cycle of centrifugation was carried out on plasma (13,400 rpm for 2 min). Platelet-free plasma samples were stored at −80 °C to ensure sufficient MPs, which were then captured by immobilized annexin V. In brief, biotinylated annexin V (annexin VBi; Roche Diagnostics, Mannheim, Germany) was bound to streptavidin-coated 96-well microtitration plates (Roche Diagnostics, Mannheim, Germany). After washing the plates three times with TBS-Ca^2+^ (50 mM Tris buffer, pH 7.5 containing 120 mM NaCl, 2.7 mM KCl, and 1 mM CaCl_2_), 300 μL of platelet-free plasma was thawed and a thrombin inhibitor and factor Xa inhibitor (Merck, Darmstadt, Germany) were added to the microwells. Then, 100 μL of platelet-free plasma was incubated for 30 min at 37 °C (duplicate microwells). After four washes, the anionic phospholipid (PhP) content was determined by a classic pro-thrombinase assay. The results are reported as nanoMoles (nM) phosphatidylserine equivalents (nM PS) according to Pigault et al. [22].

#### 2.1.2. Pro-Thrombinase-Induced Clotting Time Assay (PiCT)

The immobilized MPs were incubated in a final volume of 150 μL with factor Va (250 pM), factor Xa (9.3 pM), prothrombin (0.7 μM), and CaCl_2_ (saturated solution) for 15 min at 37 °C (all reagents from Sigma Aldrich, USA). Then, 1.5 mM Chromozym TH (Roche Diagnostics, Mannheim, Germany) was added as a chromogenic substrate for thrombin and the solution was incubated for another 4 min. The chromogenic substrate was cleaved by thrombin. The color change was measured photometrically (405 nm) using a microtitration plate reader (Thermo Fisher Scientific Inc.,Waltham, MA, USA) equipped with kinetic software [22]. The color development reaction followed a Michaelis–Menten kinetic and was stopped after 4 min by the addition of EDTA (5 mM, Merck, Kenilworth, New Jersey, USA). Velocity was reported in seconds (sec).

#### 2.1.3. Phospholipids (PLPs)

The PLPs were quantified according to a modified Stewart assay (using 100 µL of platelet-free plasma). The Stewart assay was based on the ability of PLPs to form a complex with ammonium ferrothiocyanate. A solution was prepared by dissolving 13.52 g of ferric chloride hexahydrate and 15.2 g of ammonium thiocyanate (both from Sigma Aldrich) in 0.5 L of water. The solution was stable at room temperature for several months. A PLP (Sigma Aldrich) calibration standard of 0.1 mg/mL was prepared in chloroform and a six-point calibration curve was performed using chloroform as the solvent, bringing the final volume to 2 mL (0–1 mL of PLP standard in 2 mL of chloroform). Finally, 2 mL of ferrothiocyanate solution was added to all six standards, prepared in glass tubes. The tubes were vortexed for 20 s and then centrifuged for 5 min at 1000 rpm, the lower layer then being removed using a Pasteur pipette. Test samples were similarly prepared. The optical density (OD) of both of the standards and samples was read at 485 nm using a Shimadzu Recording Spectrophotometer UV-2401PC. Test sample concentrations were found by comparing them with the six-point standard curve built using external standards.

#### 2.1.4. Oxidative Stress Measures (MDA, 4-HNE, and TBARSs)

The methodology for determining MDA and 4-HNE has been described previously [14]. MDA and 4-HNE were determined as a complex with thiobarbituric acid (TBA). Briefly, 125 mL of TBA (0.25 g in 50 mL H_2_O), 150 mL of HPLC-grade H_2_O, and 325 mL of phosphoric acid (H_3_PO_4_, 0.15M) were added to 100 mL of plasma (in EDTA). The sample was incubated at 90 °C for 1 h for the MDA derivatization with TBA and at 45 °C for 1 h for 4-HNE derivatization. After the incubations, duplicate samples were placed in ice, centrifuged at 15,000× *g* for 10 min, and syringe-filtered (0.45 mm, Superchrom srl, Milan, Italy). Twenty microliters of the sample was then successively analyzed by HPLC (Perkin-Elmer) using a Lichrospher100 RP-18 (250 mm–4 mm) column (Superchrom srl, Milan, Italy) equipped with a 785A absorbance detector and a LC240 fluorescence detector (532 nm exc., 553 nm em.). The mobile phase contained 200 mL of methanol and 300 mL of phosphate-buffered saline (50 mM, pH 7.4). A standard curve was generated using commercial standards from Cayman Chemical Company for 4-HNE (Ann Arbor, MI, USA) and 1,1,3,3-tetraethoxypropane (Merck, Darmstadt, Germany) for MDA.

#### 2.1.5. Galectin-3 (Gal-3)

The pre-coated 96-well plate solid-phase sandwich enzyme-linked immunosorbent assay (ELISA; Waltham, MA, USA) was used for the analysis of human Gal-3 in plasma. A target-specific antibody was used to pre-coat the wells of the supplied microplate, and a sandwich was formed by the addition of the second (detector) antibody and a substrate solution that reacted with the enzyme–antibody–target complex to produce a measurable signal. The intensity of this signal was directly proportional to the concentration of the target present in the original specimen and was recorded using an ELISA microplate reader (ThermoFisher Scientific). The limit of detection (LOD) for Gal-3, defined as the concentration resulting in an absorption significantly higher than the absorption of the dilution medium (mean plus two standard deviations), was certified to be 0.29 ng/mL (mean of six independent assays) and the limit of quantification (LOQ) was 0.47 ng/mL. Fresh samples of plasma (citrate) were collected and centrifuged. Samples containing a visible precipitate were clarified prior to use in the assay. Samples were aliquoted and stored at −80 °C (Piardi, Italy) to avoid the loss of bioactive human GAL-3 prior to analysis. Prior to the assay, frozen samples were brought to room temperature slowly and mixed gently using a mechanical laboratory agitator (FALC Instruments S.r.l., Treviglio, Bergamo, Italy). To carry out the GAL-3 analysis, several materials and consumables were used, including Eppendorf Research^®^ Plus adjustable single channel micropipettes with disposable tips (5–500 μL and 200–1000 μL), Eppendorf Research^®^ Plus adjustable multichannel micropipettes with disposable tips (50–300 μL), multichannel micropipette reservoirs, multichannel wash bottles, and generic consumables for laboratory analysis. HPLC-grade water was produced in the laboratory using a Milli-Q^®^ system. All reagents of the kit were prepared according to the ELISA product manual.

Briefly, each purified sample (50 µL), standard, and blank were assayed in duplicate. The microwell strips were washed twice with 400 μL of wash buffer and 100 μL of sample diluent was added to all microwells dedicated to the standard curve. A total of 100 μL of the prepared standard (concentration = 60 ng/mL) was dispensed into wells A1 and A2. The contents of wells A1 and A2 were mixed by repeated aspiration and ejection (concentration of standard 1, S1 = 30 ng/mL), and 100 μL was transferred to wells B1 and B2, respectively. We continued this procedure five times, creating two rows of galectin-3 standard dilutions, ranging from 30 to 0.47 ng/mL. The microplate was then covered and incubated for 1 h at room temperature (18–25 °C) on a microplate shaker.

Then, 100 μL the Horseradish Peroxidase (HRP)-conjugate was added to all microwells and the microplate was covered and incubated for 1 h at room temperature on a microplate shaker. The microplate was emptied and washed four times with wash buffer and 100 μL of 3,3′,5,5′-Tetramethylbenzidine (TMB)substrate solution was immediately added to all wells. The microplate was incubated for approximately 30 min at room temperature, then 100 μL of stop solution was added to all wells. Finally, the microplate was read at 450 nm using a Thermo Fisher ELISA microplate reader.

#### 2.1.6. Superoxide Dismutase Analysis

According the instructions of the Cayman Chemical Superoxide Dismutase Assay Kit (Ann Arbor, MI, USA), all reagents and standards provided in the kit were diluted and prepared for the microwell plate. Briefly, serum samples were diluted 1:5 with sample buffer. Then, 200 µL of diluted radical detector and 10 µL of seven standard concentrations per well were added (from A1 to G1, double wells). For the sample wells, 200 µL of diluted radical detector was added. Diluted xanthine oxidase (20 µL) was added very quickly to all wells, then the plate was shaken for a few minutes and covered with the specific Cayman Chemical film. The plate was incubated for 30 min at room temperature and absorbance was read at 450 nm using a Thermo Fisher ELISA microplate reader.

### 2.2. Statistical Analysis

Continuous and categorical variables are presented as median (IQR) and relative frequencies (%), respectively. Clinical and hemodynamic variables were compared by the Mann–Whitney *U* test. In order to evaluate the effects of MPs and OxS biomarkers on the others (i.e., PLPs, PiCT, and Gal-3), the non-linear regression function was used, graphically represented by regression curves. Only for Gal-3 did we use the linear regression function. Statistical significance was conventionally defined as *p* < 0.05. Statistical analyses were performed using statistical software SPSS for Windows (Statistical Package for the Social Science, version 21.0; SPSS Inc., Chicago, IL, USA).

## 3. Results

The demographic and clinical characteristics of the cases and controls are summarized in Table 1.

MPs were found to be not significantly different between the VTE patients compared to the controls. Interestingly, PLPs were found to be higher in patients with VTE than in controls, whilst PiCT was reduced. Overall, the OxS biomarkers were found to be higher in patients compared to controls. The Gal-3 value was higher in patients with VTE than in controls, while SOD was reduced in VTE patients compared to controls. (Table 2)

No differences in the laboratory values were found between patients according to the type of thrombotic disease, with the exception of SOD, which was significantly higher in patients with DVT than in those with PE. (Table 2)

**Figure 1:**
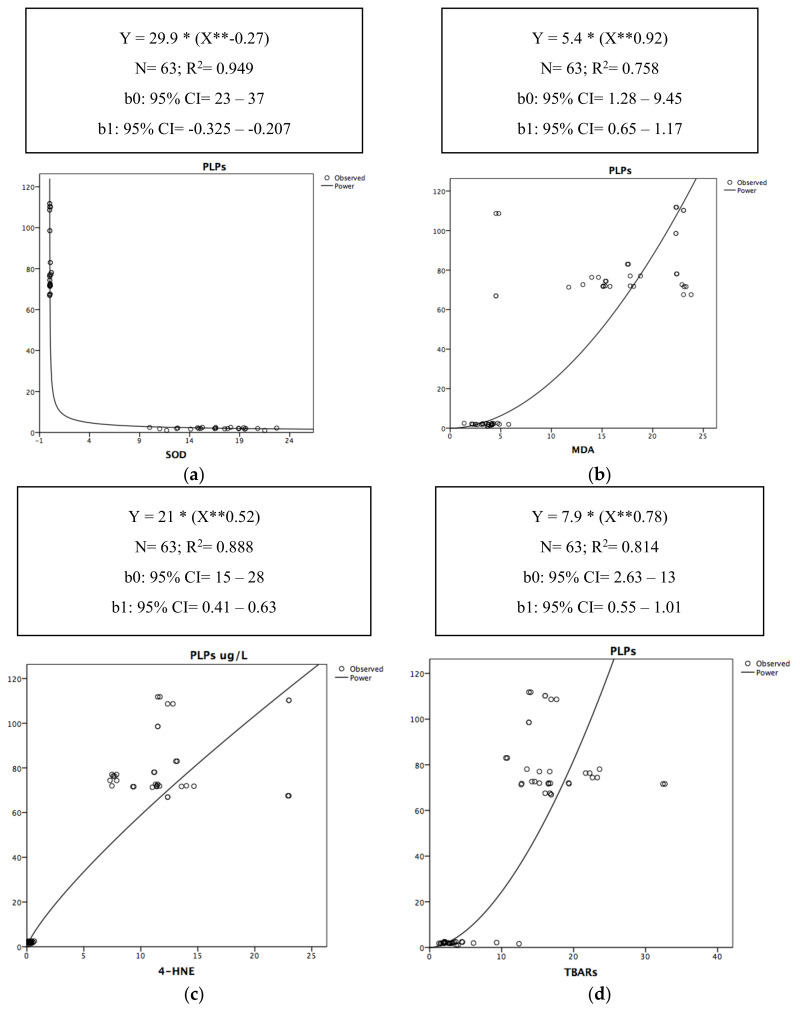
(**a**–**d**) Non-linear scatter diagrams of determination between PLPs and SOD (**a**), MDA (**b**), 4-HNE (**c**), TBARs (**d**). R^2^ = the value of R^2^ (in terms of percentage) expresses the percentage of the variability of the outcome (PLPs) that can be explained by the predictor. *: the asterisk in the curve equation equals to the “×” sign of multiplication. **: the two asterisks in the curve equation equals to “X” squared.

In Figure 1, the relationships of the determination between PLPs and each of the surrogate OxS biomarkers (SOD, MDA, 4-HNE, and TBARs) are graphically represented by a power-type regression.

Figure 1a–d thus highlights the moment when SOD, MDA, 4-HNE, and TBARs impacted the PLP level. Therefore, PLPs decreased with the increase in SOD, starting from approximately 1 U/mL (Figure 1a), with the increase of both MDA and TBARs starting from approximately 5 μM/L (Figure 1b,d). PLPs increased simultaneously with the increase in 4-HNE (Figure 1c).

**Figure 2:**
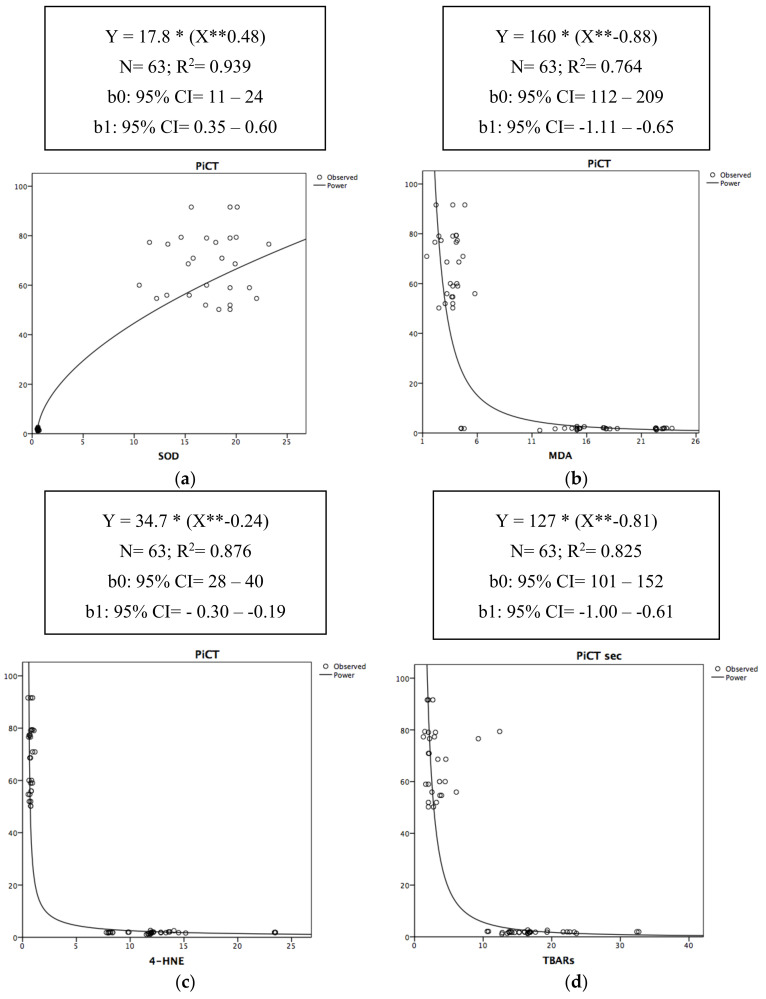
(**a**–**d**) Non-linear scatter diagrams of determination between PiCT and SOD (**a**), MDA (**b**), 4-HNE (**c**), TBARs (**d**). R^2^ = the value of R^2^ (in terms of percentage) expresses the percentage of the variability of the outcome (PiCT) that can be explained by the predictor. *: the asterisk in the curve equation equals to the “×” sign of multiplication. **: the two asterisks in the curve equation equals to “X” squared.

In Figure 2, the relationships of the determination between PiCT and each of the surrogate OxS biomarkers (SOD, MDA, 4-HNE, and TBARs) are graphically represented by a power-type regression.

Figure 2a–d thus highlights the moment when SOD, MDA, 4-HNE, and TBARs impacted the PiCT level. Therefore, PiCT increased with the increase in SOD (Figure 1a); conversely, it decreased with the increase in MDA, 4-HNE, and TBARs, starting from approximately 1 μM/L (Figure 2b–d).

**Figure 3:**
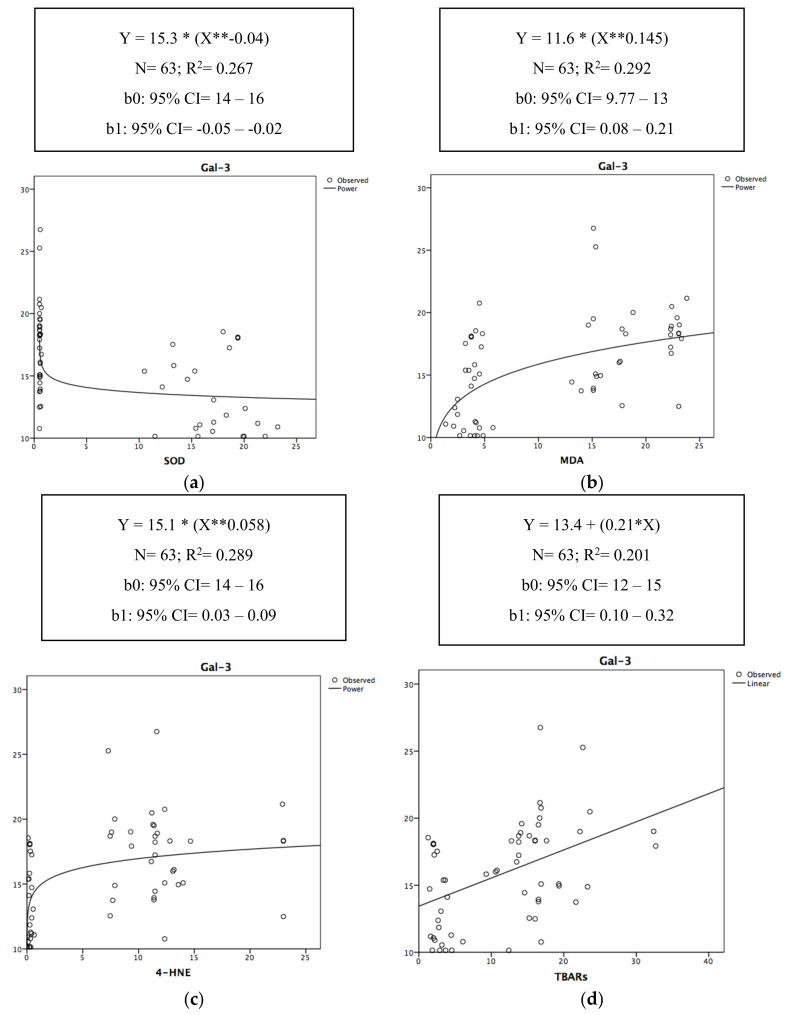
(**a**–**d**) Non-linear scatter diagrams of the determination between Gal-3 and SOD (**a**), MDA (**b**), 4-HNE (**c**), TBARs (**d**). R^2^ = the value of R^2^ (in terms of percentage) expresses the percentage of the variability of the outcome (Gal-3) that can be explained by the predictor. *: the asterisk in the curve equation equals to the “×” sign of multiplication. **: the two asterisks in the curve equation equals to “X” squared.

In Figure 3, the relationship of the determination between Gal-3 and each of the surrogate OxS biomarkers (SOD, MDA, 4-HNE, and TBARs) are graphically represented by a power-type regression, except for TBARs, which is linear. 

Figure 3a–d thus highlights the moment when SOD, MDA, 4-HNE, and TBARs impacted the Gal-3 level. Therefore, Gal-3 decreased with the increase in SOD (Figure 3a); conversely, it increased with the increase in MDA, 4-HNE, and TBARs (Figure 3b–d).

**Figure 4:**
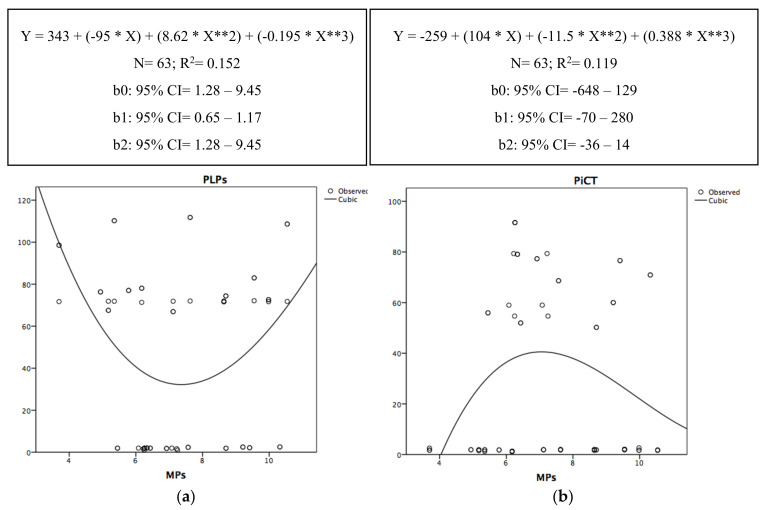
Non-linear scatter diagrams of the determination between PLPs (**a**), PiCT (**b**), and MPs. R^2^ = Table 2. (in terms of percentage) expresses the percentage of the variability of the outcome (PLPs and PiCT) that can be explained by the predictor (MPs). *: the asterisk in the curve equation equals to the “×” sign of multiplication. **: the two asterisks in the curve equation equals to “X” squared.

In Figure 4a,b, the relationship of the determination between PLPs, PiCT, and MPs are graphically represented by a cubic-type regression (non-linear regression). The peak of the parabola (Figure 4a) thus highlights the moment when the MPs impacted the PLP level. Therefore, PLPs decreased with the increase in MPs until the level of MPs was approximately 7 nM PHS equivalent, after which, PLPs increased simultaneously to MPs.

The peak of the parabola (Figure 4b) thus highlights the moment when MPs impacted the PiCT level. Thus, PiCT increased with the increase in MPs until the level of MPs was approximately 7 nM PHS equivalent, after which, PLPs dropped.

## 4. Discussion

It is known that MPs favor clotting because they have a high procoagulant capability via increasing the assembly of procoagulative factors. To explain this procoagulative activity, we must take into account that the surface of MPs contains both clot proteins and phosphatidylserine. In turn, clot proteins greatly increase the activated complex of the activated factors VIII/IX and V/X of the coagulative cascade [23,24]. Furthermore, MPs release TF, a potent initiator of procoagulant activity in vitro. TF dramatically increases procoagulant activity and the risk of thromboembolic events. To the best of our knowledge, platelet-derived MPs are closely linked to activated coagulative factor complexes; therefore, MPs play a potential role in accelerating thrombin generation. It has been noted that the hematic stasis, which occurs with an altered venous circulation, leads to hypoperfusion and hypoxia. Both of these situations increase reactive oxygen species (ROS) generation, activate OxS, and promote cell death. Conclusively, these conditions lead to a hypercoagulative status. OxS is defined as an imbalance between the oxidant and antioxidant systems and results from either the overproduction of free oxygen radicals or the insufficiency of antioxidant mechanisms. It has been demonstrated the generation of ROS and an increased plasma level of other oxidants are involved in venous thrombotic diseases. Few studies have been conducted to assess OxS in thrombotic diseases of the venous circulation, although high levels of myeloperoxidase, MDA, and 4-HNE have been demonstrated in VTE [16,25].

The results from a previous study demonstrated a direct relationship between increased values (quintiles) of Gal-3 and the rate of VTE per 1000 person/years, suggesting that Gal-3 is associated with the incidence of VTE [16]. Platelet-derived MPs are closely linked to activated coagulative factor complexes; therefore, MPs play a key role in thrombin generation [26,27]. Additionally, we must take into account that Gal-3 levels may mimic the role played by inflammation in VTE pathogenesis. Gal-3 contributes to the pro-inflammatory pathway in VTE through interleukin-6 [27]. High plasma levels of interleukin-6 have been found to be pro-inflammatory in venous thrombotic diseases [28]. It is also known that the multimerization of Gal-3 seems to enhance cell-to-cell interactions [26,29]. Additionally, the exogenous administration of Gal-3 induces the secretion of pro-inflammatory cytokines such as interleukin-6 and tumor necrosis factor-alpha. It has also been demonstrated that Gal-3 interacts with a Gal-3 binding protein at the thrombus wall interface, favoring thrombosis [26].

Gal-3 is mainly found in normal adult tissues, within cell nucleus, in cell surface and in extracellular spaces as fragmented multimers after enzymatic cleavage. This last has consequences; in particular, it enhances the ability of Gal-3 to facilitate cell-to cell interactions. Both cell adhesion and inflammation are known to play a role in promoting the pathophysiologic mechanisms of venous thrombosis. In this regard, it is interesting to note that Gal-3 multimers were found in bloodstream cells, including red blood cells, leukocytes, and platelets, as well as in platelet-derived microparticles. High levels of Gal-3 highlights it as interesting marker of cell-to-cell interaction and of inflammation in venous thromboembolism.

We emphasized the role of platelets in VTE, as demonstrated by the high level of PLPs in patients with venous thrombosis. It is known that in platelet activation, antiphospholipid antibodies mark hypercoagulative conditions, characterizing diseases (e.g., antiphospholipid syndrome, disseminate intravascular coagulation, and heparin-induced thrombocytopenia) with a high risk of VTE. To investigate a potential close link between the count and diameter of platelets, their activation was analyzed in patients with the VTE, as the results from previous studies led to us considering this cell to play a role in VTE [30,31,32,33].

The results from the present study show that the OxS process is highly activated in VTE, and was found in patients diagnosed with both DVT and PE. In fact, the biomarkers MDA, 4-HNE, and TBARs as surrogates of the activated OxS process, and Gal-3 as a biomarker of bloodstream cell endorsement, were found to be higher in VTE patients than in controls. The aforementioned pathway confirms the imbalance between the antioxidant and oxidative systems in VTE, and consequently, the balance moved towards the pro-oxidative pathway. The lower levels of SOD found in VTE patients compared to controls confirm the reduced capacity of the redox system. Our results concerning MPs, PiCT, and PLPs may help to highlight two pathophysiologic mechanisms in VTE: We demonstrated a difference in the activation of platelets, and we also demonstrated accelerated thrombin generation. Furthermore, we demonstrated the occurrence of OxS in patients with VTE. Because these mechanisms favor clot formation, we might consider these mechanisms as multifaceted mechanisms in the pathophysiology of VTE. Although previous studies have assessed OxS, platelets, and platelet-derived MPs separately, to the best of our knowledge, this is the first study considering OxS and MPs together in VTE. Our results led us to hypothesize that OxS and degranulation of the platelet membrane are concomitant mechanisms in the pathophysiology of VTE. We hope that the findings of our study will be helpful in improving the knowledge of the pathophysiology of VTE and in promoting further research into the pathways and effective therapeutic options for VTE patients.

## 5. Strengths and Limitations

Our study assessed platelet microparticles, thrombin generation, and OxS, which are not commonly considered to be involved in the pathophysiology of VTE. The limited number of VTE patients affected the study’s sample size, and it could be considered as a limitation of this study. However, we want to draw attention to the low estimated frequency of both venous thromboembolic diseases in patients admitted to a single medical center compared to other vascular diseases. Furthermore, we matched a similar number of VTE patients (cases) with healthy individuals (controls) to assess differences regarding the markers.

## 6. Conclusions

Our study demonstrated that the enhanced activation of platelets is a prothrombotic event and demonstrated the presence of OxS in VTE. We hope that our findings will encourage further studies to elucidate the mechanisms of VTE.

## Figures and Tables

**Table 1:** Table shows the age and clinical characteristic of the venous thromboembolism (VTE) patients and of controls.

	Cases	Controls
*N*	36	36
Males, *n (%)*	24 (66.7)	14 (53.8)
Median age (years) (IQR) *	64 (50–72)	55 (52–58)
Pulmonary embolism, *n (%)*	11 (30.6)	0
Deep vein thrombosis, *n (%)*	25 (69.4)	0
Previous VTE, *n (%)*	10 (27.7)	0
No previous VTE, *n (%)*	20 (55.5)	0
Heart disease, *n (%)*	10 (27.7)	0
Thrombophilia, *n (%)*	12 (33.3)	0
Type 2 diabetes, *n (%)*	7 (19.4)	0
Overweight, *n (%)*	10 (27.7)	0

* IQR: interquartile range (25th–75th).

**Table 2:** The median values of different measured biomarkers in overalls case and in controls, as well as in patients having deep vein thrombosis (DVT) and pulmonary embolism (PE). We performed statistical comparison (Mann–Whitney *U* test) concerning the overall cases vs. controls and DVT vs. PE.

	Cases Median (IQR) *	Controls Median (IQR) *	DVT Median (IQR) *	PE Median (IQR) *	*p*-Value ^a^	*p*-Value ^b^
**MDA** (µM/L)	17.7 (15.1–22.4)	3.8 (3.0–4.1)	17.8 (15.3–22.4)	14.7 (4.5–23.1)	0.00	0.2
**4-HNE** (µM/L)	11.5 (9.4–13.1)	0.3 (0.2–0.4)	11.5 (11.2–13.1)	12.4 (7.7–14.7)	0.00	0.49
**TBARs** (µM/L)	16.5 (14.2–19.3)	2.7 (2.0–3.8)	16.5 (13.8–19.3)	16.9 (16.0–21.7)	0.00	0.15
**Gal-3** (ng/mL)	18.3 (14.9–19.1)	12.7 (10.7–17.3)	18.2 (14.9–19.6)	18.3 (13.7–18.7)	0.00	0.45
**SOD** (U/mL)	0.03 (0.01–0.07)	17.05 (14.63–19.02)	0.07 (0.03–0.08)	0.01 (0.01–0.02)	0.00	0.00
**MPs** (nM PHS equivalent)	7.1 (5.3–8.9)	7.0 (6.3–8.8)	7.1 (5.2–8.7)	7.1 (5.4–10.5)	0.54	0.64
**PLPs** (µg/mL)	74.4 (71.8–86.9)	2.0 (1.9–2.2)	74.4 (71.9–83.0)	76.3 (71.6–108.6)	0.00	0.92
**PiCT** (0.00 s)	1.9 (1.7–1.9)	68.7 (55.6–77.7)	1.9 (1.7–2.0)	1.9 (1.8–2.0)	0.00	0.87

Legend. * Interquartile range (IQR; 25th–75th). ^a^ *p*-value Mann–Whitney *U* test for cases vs. controls. ^b^ *p*-value Mann–Whitney *U* test for DVT vs. PE. MDA = malondialdehyde; 4-HNE = 4-hydroxynonenale; TBARs = thiobarbituric acid reactive substances; Gal-3 = galactine-3; MPs = platelet-derived microparticles; PHS: Phosphatidyl-L-serine; PLPs = phospholipids; PiCT = pro-thrombinase-induced clotting time assay.
